# Statistical Properties of Superpositions of Coherent Phase States with Opposite Arguments

**DOI:** 10.3390/e26110977

**Published:** 2024-11-15

**Authors:** Miguel Citeli de Freitas, Viktor V. Dodonov

**Affiliations:** 1 Institute of Physics, University of Brasilia, P.O. Box 04455, Brasilia 70919-970, DF, Brazil; miguelciteli@hotmail.com; 2 International Center for Physics, University of Brasilia, Brasilia 70919-970, DF, Brazil

**Keywords:** coherent phase states, even/odd superpositions, Yurke–Stoler superpositions, squeezing, the Mandel factor, the Robertson–Schrödinger uncertainty product

## Abstract

We calculate the second-order moments, the Robertson–Schrödinger uncertainty product, and the Mandel factor for various superpositions of coherent phase states with opposite arguments, comparing the results with similar superpositions of the usual (Klauder–Glauber–Sudarshan) coherent states. We discover that the coordinate variance in the analog of even coherent states can show the most strong squeezing effect, close to the maximal possible squeezing for the given mean photon number. On the other hand, the Robertson–Schrödinger (RS) uncertainty product in superpositions of coherent phase states increases much slower (as function of the mean photon number) than in superpositions of the usual coherent states. A nontrivial behavior of the Mandel factor for small mean photon numbers is discovered in superpositions with unequal weights of two components. An exceptional nature of the even and odd superpositions is demonstrated.

## 1. Introduction

Since the beginning of 1960s, one of the main tools in quantum mechanics and quantum optics is the Klauder–Glauber–Sudarshan coherent state (CS) [1,2,3],

(1) $$|\alpha \rangle =\exp\left(-{\left|\alpha \right|}^{2}/2\right)\sum_{n=0}^{\infty }\frac{\alpha^{n}}{\sqrt{n!}}|n\rangle ,$$

where the Fock state |n〉 [4] is the eigenstate of the number operator ${\hat{a}}^{†}\hat{a}$: ${\hat{a}}^{†}\hat{a}|n\rangle =n|n\rangle$, and $\alpha =\left|\alpha \right|e^{i\varphi }$ may be an arbitrary complex number. Here, ${\hat{a}}^{†}$ and $\hat{a}$ are the bosonic creation and annihilation operators satisfying the canonical commutation relation $[\hat{a},{\hat{a}}^{†}]=1$. The coherent state (1) is the eigenstate of the annihilation operator $\hat{a}$:

(2) $$\hat{a}|\alpha \rangle =\alpha |\alpha \rangle .$$

Among numerous generalizations of the state (1) (see, e.g., reviews [5,6,7,8,9,10,11]), we distinguish here the family of the *coherent phase states* (CPS) [12,13,14,15,16,17,18,19,20,21] (called also as “harmonious states” [22] and “pseudothermal states” [23]),

(3) $$|\varepsilon \rangle =\sqrt{1-{\left|\varepsilon \right|}^{2}}\sum_{n=0}^{\infty }\varepsilon^{n}|n\rangle ,\;\varepsilon =\left|\varepsilon \right|e^{i\varphi },\;\left|\varepsilon \right|<1,$$

introduced as eigenstates of the Susskind–Glogower exponential phase operator [24],

(4) $${\hat{E}}_{-}|\varepsilon \rangle =\varepsilon |\varepsilon \rangle ,\;{\hat{E}}_{-}={\left(\hat{a}{\hat{a}}^{†}\right)}^{-1/2}\hat{a},\;{\hat{E}}_{+}={\hat{E}}_{-}^{†},$$

(5) $${\hat{E}}_{+}|n\rangle =|n+1\rangle ,\;{\hat{E}}_{-}|n\rangle =\left(1-\delta_{n0}\right)|n-1\rangle .$$

Another important family of quantum states, which turned out useful for many applications of quantum mechanics and quantum information, consists of normalized superpositions of coherent states |α〉 and |−α〉 of the form [7]

(6) $${|\Psi \rangle }_{r\alpha }=N_{r\alpha }\left(|\alpha \rangle +r|-\alpha \rangle \right),\;r=\left|r\right|e^{i\theta },\;N_{r\alpha }^{2}={\left[1+{\left|r\right|}^{2}+2Re\left(r\right)\chi_{\alpha }\right]}^{-1},$$

(7) $$\chi_{\alpha }=\langle -\alpha |\alpha \rangle =\exp\left(-{2|\alpha |}^{2}\right).$$

The most interesting choices of parameter *r* are related to the values r=±1, which correspond to the *even and odd coherent states* introduced by Dodonov, Malkin, and Man’ko [25]. The choice r=i was made by Yurke and Stoler [26].

In this paper, we study the properties of analogs of states (6), where the usual coherent states are replaced with the *coherent phase states* (3):

(8) $${|\Psi \rangle }_{r\varepsilon }=N_{r\varepsilon }\left(|\varepsilon \rangle +r|-\varepsilon \rangle \right),\;N_{r\varepsilon }^{2}={\left[1+{\left|r\right|}^{2}+2Re\left(r\right)\chi_{\varepsilon }\right]}^{-1},$$

(9) $$\chi_{\varepsilon }=\langle -\varepsilon |\varepsilon \rangle =\frac{1-{\left|\varepsilon \right|}^{2}}{1+{\left|\varepsilon \right|}^{2}}.$$

If r=exp(iθ), the normalization factor assumes the form

(10) $$N_{\theta \varepsilon }^{2}=\frac{1+{\left|\varepsilon \right|}^{2}}{4\left[\cos^{2}\left(\theta /2\right)+{\left|\varepsilon \right|}^{2}\sin^{2}\left(\theta /2\right)\right]},$$

with the following special values:

(11) $$N_{0\varepsilon }^{2}=\frac{1+{\left|\varepsilon \right|}^{2}}{4},\;N_{\pi \varepsilon }^{2}=\frac{1+{\left|\varepsilon \right|}^{2}}{{4|\varepsilon |}^{2}},\;N_{\pi /2,\varepsilon }^{2}=\frac{1}{2}.$$

The superpositions (6) and (8) are eigenstates of squares of the corresponding annihilation operators:

$${\hat{a}}^{2}{|\Psi \rangle }_{r\alpha }=\alpha^{2}{|\Psi \rangle }_{r\alpha },\;{\hat{E}}_{-}^{2}{|\Psi \rangle }_{r\varepsilon }=\varepsilon^{2}{|\Psi \rangle }_{r\varepsilon }.$$

Even and odd superpositions of the CPS have the following expansions in the Fock basis:

(12) $${|\Psi \rangle }_{1\varepsilon }=\sqrt{1-{\left|\varepsilon \right|}^{4}}\sum_{n=0}^{\infty }\varepsilon^{2n}|2n\rangle ,\;{|\Psi \rangle }_{-1\varepsilon }={\left|\varepsilon \right|}^{-1}\sqrt{1-{\left|\varepsilon \right|}^{4}}\sum_{n=0}^{\infty }\varepsilon^{2n+1}|2n+1\rangle .$$

The states (12) were considered in paper [27]. However, their statistical properties were studied in that paper for moderate values of parameter ε only, while the most interesting features can be observed in the limit |ε|→1, as will be shown in the following sections. Truncated series were considered in [28] but only for small truncation numbers.

Functions $\chi_{\alpha }$ and $\chi_{\varepsilon }$ characterize the overlaps between the wave functions with opposite arguments. In the case of usual coherent states, these Gaussian functions are rather narrow and well localized. For this reason, their scalar product decreases exponentially when the distance between two components of the superposition increases. On the other hand, the non-Gaussian wave functions of the coherent phase states are rather wide so that the related overlap integral decays much more slowly as function of parameter ε. The goal of this paper is to study what the physical consequences of these differences are. For example, it is known that usual coherent states have no squeezing of the canonical position and momentum operators (we assume ħ=1),

(13) $$\hat{x}=\left(\hat{a}+{\hat{a}}^{†}\right)/\sqrt{2},\;\hat{p}=\left(\hat{a}-{\hat{a}}^{†}\right)/\left(i\sqrt{2}\right),$$

for any value of the complex parameter α. On the other hand, we discovered recently [21] that strong squeezing is possible for the CPS with φ=π/2 when |ε|→1. Therefore, it is interesting to know whether the degree of squeezing can be enhanced even more in the states (8), remembering that small squeezing was observed in the even coherent states [29]. This question is discussed in Section 3. All necessary formulas for the mean values, variances, and covariances

$$\sigma_{A}\equiv \langle {\hat{A}}^{2}\rangle -{\langle \hat{A}\rangle }^{2},\;\sigma_{AB}\equiv \frac{1}{2}\langle \hat{A}\hat{B}+\hat{B}\hat{A}\rangle -\langle \hat{A}\hat{B}\rangle ,$$

for the pair of operators $\left(\hat{x},\hat{p}\right)$ in the states (6) and (8) are derived in Section 2.

In Section 4, we compare the Robertson–Schrödinger uncertainty relations [30,31] in the states (6) and (8). It is known that the Heisenberg product of uncertainties attains the minimal possible value for all usual coherent states. On the other hand, its generalization – the Robertson–Schrödinger uncertainty combination – slowly increases logarithmically as |ε|→1 in the CPS [21]. Therefore, it is interesting to know the behavior of the RS uncertainty product in the superposition states.

In Section 5, we compare the Mandel factor for two families of superposition states. This factor equals zero identically for all coherent states. On the other hand, it can be negative for usual odd coherent states. Now, we study the dependence of this factor on ε and *r* in the superpositions of coherent phase states.

## 2. Mean Values and Variances

The mean value of any operator $\hat{A}$ in the superposition (8) is a sum of four terms:

(14) $${\langle \hat{A}\rangle }_{r\varepsilon }=N_{r\varepsilon }^{2}\left(\langle \varepsilon |\hat{A}|\varepsilon \rangle +{\left|r\right|}^{2}\langle -\varepsilon |\hat{A}|-\varepsilon \rangle +r\langle \varepsilon |\hat{A}|-\varepsilon \rangle +r^{*}\langle -\varepsilon |\hat{A}|\varepsilon \rangle \right).$$

Taking $\hat{A}=\hat{a}$ and $\hat{A}={\hat{a}}^{†}$, we obtain the following expressions for mean values of the creation and annihilation operators:

(15) $${\langle \hat{a}\rangle }_{r\varepsilon }=N_{r\varepsilon }^{2}\varepsilon \left(1-{\left|\varepsilon \right|}^{2}\right)\left[S_{+1}\left(|\varepsilon |\right)\left(1-{\left|r\right|}^{2}\right)+S_{-1}\left(|\varepsilon |\right)\left(r^{*}-r\right)\right],$$

$${\langle {\hat{a}}^{†}\rangle }_{r\varepsilon }={\langle \hat{a}\rangle }_{r\varepsilon }^{*},$$

(16) $$S_{\pm 1}\left(|\varepsilon |\right)=\sum_{n=0}^{\infty }{\left(\pm {\left|\varepsilon \right|}^{2}\right)}^{n}\sqrt{n+1}.$$

Mean values of the quadrature components (13) are as follows:

(17) 〈x^〉rε=2Nrε21−|ε|2S+1(|ε|)1−|r|2Re(ε)+2S−1(|ε|)Im(r)Im(ε),

(18) 〈p^〉rε=2Nrε21−|ε|2S+1(|ε|)1−|r|2Im(ε)−2S−1(|ε|)Im(r)Re(ε).

The most simple expressions arise for the “equal weights” superpositions with |r|=1. Then, ${\langle \hat{x}\rangle }_{r\varepsilon }={\langle \hat{p}\rangle }_{r\varepsilon }=0$ if Im(r)=0, i.e., for even and odd superpositions, for all values of ε. On the other hand, these mean values can be nonzero for the Yurke–Stoler superpositions with r=i.

For the second-order mean values, Equation (14) leads to the following formulas:

(19) $${\langle {\hat{a}}^{2}\rangle }_{r\varepsilon }=N_{r\varepsilon }^{2}\varepsilon^{2}\left(1-{\left|\varepsilon \right|}^{2}\right)\left[S_{+2}\left(|\varepsilon |\right)\left(1+{\left|r\right|}^{2}\right)+S_{-2}\left(|\varepsilon |\right)\left(r^{*}+r\right)\right],$$

$${\langle {\hat{a}}^{†2}\rangle }_{r\varepsilon }={\langle {\hat{a}}^{2}\rangle }_{r\varepsilon }^{*},$$

(20) $$S_{\pm 2}\left(|\varepsilon |\right)=\sum_{n=0}^{\infty }{\left(\pm {\left|\varepsilon \right|}^{2}\right)}^{n}\sqrt{\left(n+1)(n+2\right)}.$$

The mean number of quanta, ${\langle {\hat{a}}^{†}\hat{a}\rangle }_{r\varepsilon }$, can be calculated with the aid of Equation (14) and formulas

$$\sum_{n=0}^{\infty }x^{n}={\left(1-x\right)}^{-1},\;\sum_{n=0}^{\infty }nx^{n}=x\frac{d}{dx}\sum_{n=0}^{\infty }x^{n}=x{\left(1-x\right)}^{-2}.$$

The result is

(21) $${\langle {\hat{a}}^{†}\hat{a}\rangle }_{r\varepsilon }=\frac{N_{r\varepsilon }^{2}{\left|\varepsilon \right|}^{2}}{1-{\left|\varepsilon \right|}^{2}}\left[1+{\left|r\right|}^{2}-2Re\left(r\right){\left(\frac{1-{\left|\varepsilon \right|}^{2}}{1+{\left|\varepsilon \right|}^{2}}\right)}^{2}\right].$$

In particular,

(22) $${\langle {\hat{a}}^{†}\hat{a}\rangle }_{ev}=\frac{{2|\varepsilon |}^{4}}{1-{\left|\varepsilon \right|}^{4}},\;{\langle {\hat{a}}^{†}\hat{a}\rangle }_{od}=\frac{1+{\left|\varepsilon \right|}^{4}}{1-{\left|\varepsilon \right|}^{4}},\;{\langle {\hat{a}}^{†}\hat{a}\rangle }_{YS}=\frac{{\left|\varepsilon \right|}^{2}}{1-{\left|\varepsilon \right|}^{2}}.$$

Note that, as a matter of fact, three apparently different expressions in Equation (22) give the same results in the limit |ε|→1.

The coordinate and momentum variances in the three special cases are as follows:

(23) $${\left.\begin{array}{c} \sigma_{x}\\ \sigma_{p} \end{array}\right\}}_{ev}=\frac{1}{2}+\frac{{2|\varepsilon |}^{4}}{1-{\left|\varepsilon \right|}^{4}}\pm \frac{1}{2}{\left|\varepsilon \right|}^{2}\left(1-{\left|\varepsilon \right|}^{4}\right)\cos\left(2\varphi \right)\left[S_{+2}\left(|\varepsilon |\right)+S_{-2}\left(|\varepsilon |\right)\right],$$

(24) $${\left.\begin{array}{c} \sigma_{x}\\ \sigma_{p} \end{array}\right\}}_{od}=\frac{1}{2}+\frac{1+{\left|\varepsilon \right|}^{4}}{1-{\left|\varepsilon \right|}^{4}}\pm \frac{1}{2}\left(1-{\left|\varepsilon \right|}^{4}\right)\cos\left(2\varphi \right)\left[S_{+2}\left(|\varepsilon |\right)-S_{-2}\left(|\varepsilon |\right)\right],$$

(25) $$\begin{array}{ccc} {\left.\begin{array}{c} \sigma_{x}\\ \sigma_{p} \end{array}\right\}}_{YS} & = & \frac{1}{2}+\frac{{\left|\varepsilon \right|}^{2}}{1-{\left|\varepsilon \right|}^{2}}-{\left|\varepsilon \right|}^{2}{\left(1-{\left|\varepsilon \right|}^{2}\right)}^{2}S_{-1}^{2}\left(|\varepsilon |\right)\\ & \pm & {\left|\varepsilon \right|}^{2}\left(1-{\left|\varepsilon \right|}^{2}\right)\cos\left(2\varphi \right)\left[S_{+2}\left(|\varepsilon |\right)+\left(1-{\left|\varepsilon \right|}^{2}\right)S_{-1}^{2}\left(|\varepsilon |\right)\right]. \end{array}$$

For r=0, we have

(26) $$\begin{array}{ccc} {\left.\begin{array}{c} \sigma_{x}\\ \sigma_{p} \end{array}\right\}}_{r=0} & = & \frac{1}{2}+\frac{{\left|\varepsilon \right|}^{2}}{1-{\left|\varepsilon \right|}^{2}}-{\left|\varepsilon \right|}^{2}{\left(1-{\left|\varepsilon \right|}^{2}\right)}^{2}S_{+1}^{2}\left(|\varepsilon |\right)\\ & \pm & {\left|\varepsilon \right|}^{2}\left(1-{\left|\varepsilon \right|}^{2}\right)\cos\left(2\varphi \right)\left[S_{+2}\left(|\varepsilon |\right)-\left(1-{\left|\varepsilon \right|}^{2}\right)S_{+1}^{2}\left(|\varepsilon |\right)\right]. \end{array}$$

In all four special cases, the coordinate variances attain minimal values for φ=π/2. The explicit formulas in this case are as follows:

(27) $$\sigma_{x,ev}^{min}=\frac{1}{2}+\frac{{2|\varepsilon |}^{4}}{1-{\left|\varepsilon \right|}^{4}}-\frac{1}{2}{\left|\varepsilon \right|}^{2}\left(1-{\left|\varepsilon \right|}^{4}\right)\left[S_{+2}\left(|\varepsilon |\right)+S_{-2}\left(|\varepsilon |\right)\right],$$

(28) $$\sigma_{x,od}^{min}=\frac{1}{2}+\frac{1+{\left|\varepsilon \right|}^{4}}{1-{\left|\varepsilon \right|}^{4}}-\frac{1}{2}\left(1-{\left|\varepsilon \right|}^{4}\right)\left[S_{+2}\left(|\varepsilon |\right)-S_{-2}\left(|\varepsilon |\right)\right],$$

(29) $$\sigma_{x,YS}^{min}=\frac{1}{2}+\frac{{\left|\varepsilon \right|}^{2}}{1-{\left|\varepsilon \right|}^{2}}-{\left|\varepsilon \right|}^{2}\left(1-{\left|\varepsilon \right|}^{2}\right)S_{+2}\left(|\varepsilon |\right)-{2|\varepsilon |}^{2}{\left(1-{\left|\varepsilon \right|}^{2}\right)}^{2}S_{-1}^{2}\left(|\varepsilon |\right),$$

(30) $$\sigma_{x,r=0}^{min}=\frac{1}{2}+\frac{{\left|\varepsilon \right|}^{2}}{1-{\left|\varepsilon \right|}^{2}}-{\left|\varepsilon \right|}^{2}\left(1-{\left|\varepsilon \right|}^{2}\right)S_{+2}\left(|\varepsilon |\right).$$

The following expressions are obtained for the coordinate-momentum covariance:

(31) $$\sigma_{xp}^{ev}=\frac{1}{2}{\left|\varepsilon \right|}^{2}\left(1-{\left|\varepsilon \right|}^{4}\right)\sin\left(2\varphi \right)\left[S_{+2}\left(|\varepsilon |\right)+S_{-2}\left(|\varepsilon |\right)\right],$$

(32) $$\sigma_{xp}^{od}=\frac{1}{2}\left(1-{\left|\varepsilon \right|}^{4}\right)\sin\left(2\varphi \right)\left[S_{+2}\left(|\varepsilon |\right)-S_{-2}\left(|\varepsilon |\right)\right],$$

(33) $$\sigma_{xp}^{YS}={\left|\varepsilon \right|}^{2}\left(1-{\left|\varepsilon \right|}^{2}\right)\sin\left(2\varphi \right)\left[S_{+2}\left(|\varepsilon |\right)+\left(1-{\left|\varepsilon \right|}^{2}\right)S_{-1}^{2}\left(|\varepsilon |\right)\right],$$

(34) $$\sigma_{xp}^{r=0}={\left|\varepsilon \right|}^{2}\left(1-{\left|\varepsilon \right|}^{2}\right)\sin\left(2\varphi \right)\left[S_{+2}\left(|\varepsilon |\right)-\left(1-{\left|\varepsilon \right|}^{2}\right)S_{+1}^{2}\left(|\varepsilon |\right)\right].$$

Approximate analytical expressions can be obtained for the series $S_{\pm 2}$ if one uses the expansion

(35) $$\begin{array}{ccc} \sqrt{\left(n+1)(n+2\right)} & = & \left(n+1\right)\sqrt{1+\frac{1}{n+1}}=\left(n+1\right)\left[1+\frac{1}{2(n+1)}-\frac{1}{8{\left(n+1\right)}^{2}}+\ldots \right]\\ & \approx & n+\frac{3}{2}-\frac{1}{8(n+1)}. \end{array}$$

The three-term approximate equality (35) is quite reasonable even for n=0, and its accuracy improves significantly for bigger values of *n*. Then, using the exact formula

$$\sum_{n=0}^{\infty }\frac{x^{n}}{n+1}=x^{-1}\int_{0}^{x}dy\sum_{n=0}^{\infty }y^{n}=-\frac{\ln(1-x)}{x},$$

we obtain the following approximate analytical expressions:

(36) $$S_{\pm 2}\left(|\varepsilon |\right)=\frac{3/2}{1\mp {\left|\varepsilon \right|}^{2}}\pm \frac{{\left|\varepsilon \right|}^{2}}{{\left(1\mp {\left|\varepsilon \right|}^{2}\right)}^{2}}\pm \frac{\ln\left(1\mp {\left|\varepsilon \right|}^{2}\right)}{{8|\varepsilon |}^{2}}.$$

### Usual Coherent States

For superposition (6) of usual coherent states, mean values have the same form (14), where ε is replaced with α. In this case, all calculations can be performed explicitly due to Equation (2) and the known scalar product $\langle \pm \alpha |\alpha \rangle =\exp\left[{\left(\pm 1-1\right)|\alpha |}^{2}\right]$. The following relations hold:

(37) $${\langle \hat{a}\rangle }_{r\alpha }=N_{r\alpha }^{2}\alpha \left[1-{\left|r\right|}^{2}+\exp\left(-{2|\alpha |}^{2}\right)\left(r^{*}-r\right)\right],$$

$${\langle {\hat{a}}^{†}\rangle }_{r\alpha }={\langle \hat{a}\rangle }_{r\alpha }^{*},$$

(38) $${\langle {\hat{a}}^{2}\rangle }_{r\alpha }=\alpha^{2},\;{\langle {\hat{a}}^{†2}\rangle }_{r\alpha }=\alpha^{*2},$$

(39) $${\langle {\hat{a}}^{†}\hat{a}\rangle }_{r\alpha }=N_{r\alpha }^{2}{\left|\alpha \right|}^{2}\left[1+{\left|r\right|}^{2}-2Re\left(r\right)\exp\left(-{2|\alpha |}^{2}\right)\right],$$

(40) $$N_{0\alpha }^{2}={\left[2\left(1+e^{-{2|\alpha |}^{2}}\right)\right]}^{-1},\;N_{\pi \alpha }^{2}={\left[2\left(1-e^{-{2|\alpha |}^{2}}\right)\right]}^{-1},\;N_{\pi /2,\alpha }^{2}=\frac{1}{2},$$

(41) $${\langle {\hat{a}}^{†}\hat{a}\rangle }_{ev}={\left|\alpha \right|}^{2}\tanh\left({\left|\alpha \right|}^{2}\right),\;{\langle {\hat{a}}^{†}\hat{a}\rangle }_{od}={\left|\alpha \right|}^{2}\coth\left({\left|\alpha \right|}^{2}\right),\;{\langle {\hat{a}}^{†}\hat{a}\rangle }_{YS}={\left|\alpha \right|}^{2},$$

(42) $${\left.\begin{array}{c} \sigma_{x}\\ \sigma_{p} \end{array}\right\}}_{ev}=\frac{1}{2}+{\left|\alpha \right|}^{2}\tanh\left({\left|\alpha \right|}^{2}\right)\pm {\left|\alpha \right|}^{2}\cos\left(2\varphi \right),$$

(43) $${\left.\begin{array}{c} \sigma_{x}\\ \sigma_{p} \end{array}\right\}}_{od}=\frac{1}{2}+{\left|\alpha \right|}^{2}\coth\left({\left|\alpha \right|}^{2}\right)\pm {\left|\alpha \right|}^{2}\cos\left(2\varphi \right),$$

(44) $${\left.\begin{array}{c} \sigma_{x}\\ \sigma_{p} \end{array}\right\}}_{YS}=\frac{1}{2}+{\left|\alpha \right|}^{2}\left(1-e^{-{4|\alpha |}^{2}}\right)\pm {\left|\alpha \right|}^{2}\cos\left(2\varphi \right)\left(1+e^{-{4|\alpha |}^{2}}\right),$$

(45) $$\sigma_{xp}^{ev}=\sigma_{xp}^{od}={\left|\alpha \right|}^{2}\sin\left(2\varphi \right),$$

(46) $$\sigma_{xp}^{YS}={\left|\alpha \right|}^{2}\sin\left(2\varphi \right)\left[1+\exp\left(-{4|\alpha |}^{2}\right)\right].$$

## 3. Squeezing

Formulas for the variances of the coordinate and momentum operators obtained in Section 2 show that the coordinate variance attains minimal values for the phase φ=π/2, both for superpositions of usual coherent states and superpositions of coherent phase states. However, concrete minimal values are quite different for these two kinds of superpositions.

### 3.1. Usual Coherent States

Equations (42)–(44) show that a moderate squeezing can be achieved for the even and Yurke–Stoler superpositions with φ=π/2:

(47) $$\sigma_{x}^{ev}=\frac{1}{2}+{\left|\alpha \right|}^{2}\left[\tanh\left({\left|\alpha \right|}^{2}\right)-1\right],\;\sigma_{x}^{YS}=\frac{1}{2}-2{\left|\alpha \right|}^{2}e^{-{4|\alpha |}^{2}}.$$

For |α|≪1, a stronger squeezing is observed for the YS-superpositions. However, the minimal absolute squeezing is attained for the even superpositions. The concrete minimal values in two superpositions are as follows:

$${\left.\sigma_{x}^{ev}\right|}_{|\alpha |\approx 0.80}\approx 0.2215,\;{\left.\sigma_{x}^{YS}\right|}_{|\alpha |=1/2}=\frac{1}{2}\left(1-e^{-1}\right)\approx 0.316.$$

**Figure 1:**
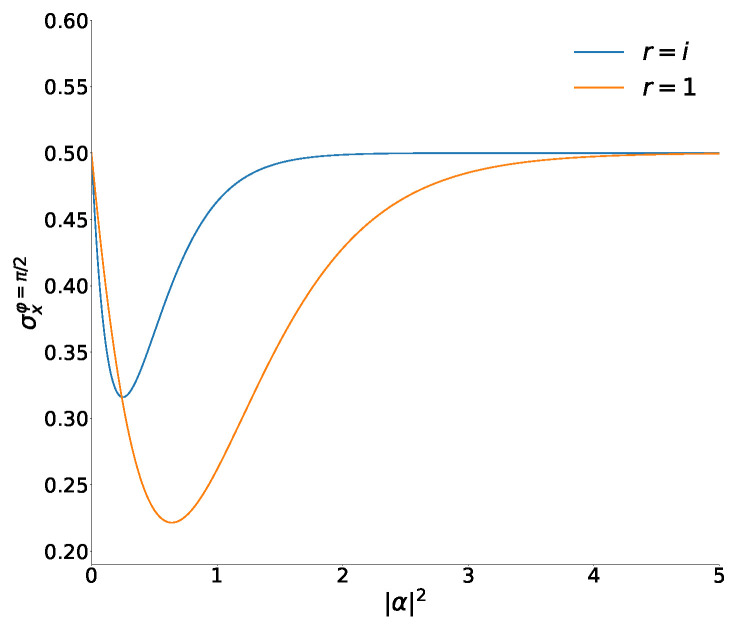
Variances $\sigma_{x}$ as functions of ${\left|\alpha \right|}^{2}$ in the even and YS superpositions of the usual coherent states with φ=π/2.

When |α|→∞ and φ=π/2, all coordinate variances tend to the asymptotic value 1/2. Two functions of Equation (47) are illustrated in Figure 1.

### 3.2. Coherent Phase States

**Figure 2:**
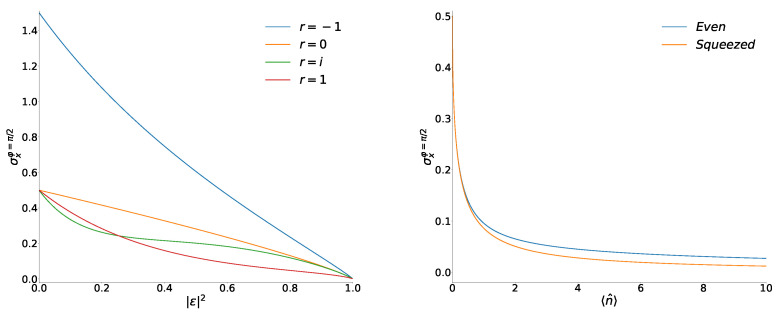
(**Left**) Variances of $\hat{x}$ as functions of ${\left|\varepsilon \right|}^{2}$ in the superpositions of the coherent phase states with φ=π/2. (**Right**) Variances of $\hat{x}$ as functions of $\langle \hat{n}\rangle$ in the even superposition of the coherent phase states with φ=π/2 compared with the variances (50) in the ideal vacuum squeezed state. All numeric results were obtained taking into account 10,000 terms in series $S_{\pm 1}$ and $S_{\pm 2}$.

On the contrary, the coordinate variances in the superpositions of coherent phase states go to zero when |ε|→1 and φ=π/2. This behavior is shown in Figure 2.

Equations (29) and (30) show that the minimal variance in the Yurke–Stoler superpositions is always smaller than in the coherent phase state (when r=0). Moreover, the squeezing in the YS-superpositions is the strongest for small values of ${\left|\varepsilon \right|}^{2}$, as can be seen in the first terms of the Taylor expansions of exact formulas (23)–(26):

$$\sigma_{x}^{r=0}\approx \frac{1}{2}-\left(\sqrt{2}-1\right){\left|\varepsilon \right|}^{2},\;\sigma_{x}^{ev}\approx \frac{1}{2}-\sqrt{2}{\left|\varepsilon \right|}^{2},\;\sigma_{x}^{YS}\approx \frac{1}{2}-\left(\sqrt{2}+1\right){\left|\varepsilon \right|}^{2}.$$

However, if |ε|>1/2, the strongest squeezing is observed in the even superpositions. The variances $\sigma_{x,YS}^{min}$ and $\sigma_{x,r=0}^{min}$ practically coincide for the values of |ε| close to unity because the sign-variable series $S_{-1}\left(|\varepsilon |\right)$ in Equation (29) remains limited when |ε|→1. The behavior of the minimal variances for small differences $1-{\left|\varepsilon \right|}^{2}$ can be described analytically with the aid of Equation (36). The leading terms of asymptotical forms of all functions (27)–(30) at ${\left|\varepsilon \right|}^{2}\to 1$ are as follows:

(48) $$\sigma_{x,r}^{min}\approx \left(1-{\left|\varepsilon \right|}^{2}\right)\left[-\frac{1}{8}\ln\left(1-{\left|\varepsilon \right|}^{2}\right)-rS\right]=\frac{\ln\left(1+\langle \hat{n}\rangle \right)-8rS}{8\left(1+\langle \hat{n}\rangle \right)},$$

(49) $$S\equiv S_{-2}{\left(|\varepsilon |\right)}_{|\varepsilon |=1}=\frac{1}{2}-\frac{1}{8}\ln\left(2\right)\approx 0.413\approx \sqrt{2}-1,\;r=\pm 1,0.$$

A thorough analysis and the comparison of (48) with a similar result of paper [21] for r=0 show that the approximation (48) is valid for extremely high values of the mean photon number $\langle \hat{n}\rangle$, namely, under the condition $\ln\left(1+\langle \hat{n}\rangle \right)\gg 1$.

Remember that the coordinate variance in the ideal pure vacuum squeezed (Gaussian) state, $\sigma_{x}=\left(1/2\right)e^{-2r}$ with $\langle \hat{n}\rangle =\sinh^{2}\left(r\right)$, as a function of the mean number of quanta $\langle \hat{n}\rangle$, is given by the known formula

(50) $$\sigma_{x}^{sqz}=\frac{1}{2}{\left[1+2\langle \hat{n}\rangle +2\sqrt{\langle \hat{n}\rangle \left(\langle \hat{n}\rangle +1\right)}\right]}^{-1}.$$

This is the minimal possible value of $\sigma_{x}$ for the fixed mean photon number $\langle \hat{n}\rangle$ [8,32,33]. On the right-hand side of Figure 2, we compare functions (27) and (50) for moderate values of the mean photon number $\langle \hat{n}\rangle$. The asymptotic form of function (50)

$$\sigma_{x}^{sqz}\approx {\left[4\left(1+2\langle \hat{n}\rangle \right)\right]}^{-1}\;for\langle \hat{n}\rangle \gg 1,$$

shows that the squeezing effect in the coherent phase states and their superpositions is only slightly weaker than in the squeezed vacuum states when $\langle \hat{n}\rangle \gg 1$.

## 4. The Robertson–Schrödinger Uncertainty Products

The Robertson–Schrödinger uncertainty relation has the form

(51) $$D\equiv \sigma_{x}\sigma_{p}-\sigma_{xp}^{2}\geq 1/4.$$

The equality D≡1/4 holds for Gaussian pure states, including the usual coherent states with arbitrary values of parameter α. Since D>1/4 for non-Gaussian states, it is interesting to know how this generalized uncertainty product depends on parameters α, ε, and *r*.

### 4.1. Usual Coherent States

Equations (42)–(46) result in the following expressions:

(52) $$D_{ev}=1/4+{\left|\alpha \right|}^{2}\tanh\left({\left|\alpha \right|}^{2}\right)-{\left|\alpha \right|}^{4}/\cosh^{2}\left({\left|\alpha \right|}^{2}\right),$$

(53) $$D_{od}=1/4+{\left|\alpha \right|}^{2}\coth\left({\left|\alpha \right|}^{2}\right)+{\left|\alpha \right|}^{4}/\sinh^{2}\left({\left|\alpha \right|}^{2}\right),$$

(54) $$D_{YS}=1/4+{\left|\alpha \right|}^{2}\left(1-e^{-{4|\alpha |}^{2}}\right)-4{\left|\alpha \right|}^{4}e^{-{4|\alpha |}^{2}}.$$

For |α|≪1, we see a small growth:

$$D_{ev}\approx 1/4+{2|\alpha |}^{8}/3,\;D_{YS}\approx 1/4+{8|\alpha |}^{6},\;D_{od}\approx 9/4+2{\left|\alpha \right|}^{8}/45.$$

If |α|≫1, the difference D−1/4 grows as ${\left|\alpha \right|}^{2}$, with exponentially small corrections. In the most general case, the asymptotic formula is

$$D\approx 1/4+\frac{{2|r|}^{2}{\left|\alpha \right|}^{2}}{1+{\left|r\right|}^{2}},\;\left|\alpha \right|\gg 1.$$

### 4.2. Coherent Phase States

The following expressions hold for the superpositions of coherent phase states:

(55) $$D_{ev}={\left(\frac{1}{2}+\frac{{2|\varepsilon |}^{4}}{1-{\left|\varepsilon \right|}^{4}}\right)}^{2}-\frac{1}{4}{\left|\varepsilon \right|}^{4}{\left(1-{\left|\varepsilon \right|}^{4}\right)}^{2}{\left[S_{+2}\left(|\varepsilon |\right)+S_{-2}\left(|\varepsilon |\right)\right]}^{2},$$

(56) $$D_{od}={\left(\frac{1}{2}+\frac{1+{\left|\varepsilon \right|}^{4}}{1-{\left|\varepsilon \right|}^{4}}\right)}^{2}-\frac{1}{4}{\left(1-{\left|\varepsilon \right|}^{4}\right)}^{2}{\left[S_{+2}\left(|\varepsilon |\right)-S_{-2}\left(|\varepsilon |\right)\right]}^{2},$$

(57) $$\begin{array}{ccc} D_{YS} & = & {\left[\frac{1}{2}+\frac{{\left|\varepsilon \right|}^{2}}{1-{\left|\varepsilon \right|}^{2}}-{\left|\varepsilon \right|}^{2}{\left(1-{\left|\varepsilon \right|}^{2}\right)}^{2}S_{-1}^{2}\left(|\varepsilon |\right)\right]}^{2}\\ & - & {\left|\varepsilon \right|}^{4}{\left(1-{\left|\varepsilon \right|}^{2}\right)}^{2}{\left[S_{+2}\left(|\varepsilon |\right)+\left(1-{\left|\varepsilon \right|}^{2}\right)S_{-1}^{2}\left(|\varepsilon |\right)\right]}^{2}, \end{array}$$

(58) $$\begin{array}{ccc} D_{r=0} & = & {\left[\frac{1}{2}+\frac{{\left|\varepsilon \right|}^{2}}{1-{\left|\varepsilon \right|}^{2}}-{\left|\varepsilon \right|}^{2}{\left(1-{\left|\varepsilon \right|}^{2}\right)}^{2}S_{+1}^{2}\left(|\varepsilon |\right)\right]}^{2}\\ & - & {\left|\varepsilon \right|}^{4}{\left(1-{\left|\varepsilon \right|}^{2}\right)}^{2}{\left[S_{+2}\left(|\varepsilon |\right)-\left(1-{\left|\varepsilon \right|}^{2}\right)S_{+1}^{2}\left(|\varepsilon |\right)\right]}^{2}. \end{array}$$

**Figure 3:**
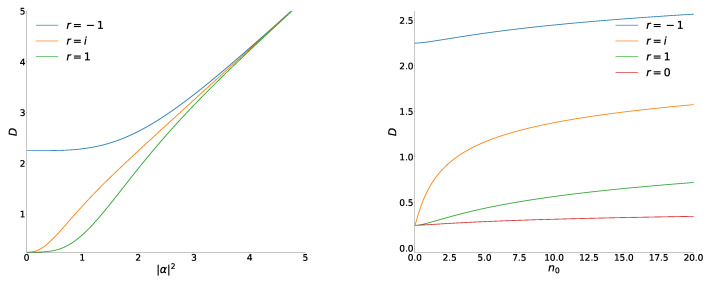
The Robertson–Schrödinger uncertainty product *D* for superpositions of coherent states (**left**) and coherent phase states (**right**) as functions of the mean number of quanta $n_{0}$ in the states with r=0. All numeric results were obtained taking into account 10,000 terms in series $S_{\pm 1}$ and $S_{\pm 2}$.

In Figure 3, we compare the RS uncertainty products in the superpositions of usual coherent states and coherent phase states, plotting these quantities as functions of the argument $n_{0}={\langle \hat{n}\rangle }_{r=0}$. This means that $n_{0}={\left|\varepsilon \right|}^{2}{/(1-|\varepsilon |}^{2})$ on the right-hand side, whereas $n_{0}={\left|\alpha \right|}^{2}$ on the left-hand side.

All expressions, (52)–(58), do not contain the phase φ of the complex arguments α and ε. This fact can be understood if one takes into account the equivalence between the phase change and time evolution. Indeed, the time evolution of states (1) and (3) of a quantum harmonic oscillator with frequency ω is reduced to the linear evolution of phases of complex parameters α and ε: φ(t)=φ(0)−ωt. On the other hand, the quantity *D* is the simplest *quantum universal invariant*, which preserves its value during the time evolution governed by *any* quadratic one-dimensional Hamiltonian [34,35].

Finding exact numeric values of functions $D_{ev}\left(|\varepsilon |\right)\equiv D_{1}\left(|\varepsilon |\right)$ and $D_{od}\left(|\varepsilon |\right)\equiv D_{-1}\left(|\varepsilon |\right)$ is rather difficult task when |ε| is close to unity. Indeed, using Equations (55)–(56), one has to find the small difference of very big numbers. The main difficulty is to calculate the slowly convergent series $S_{+2}\left(|\varepsilon |\right)$ with high precision. For example, if ${\left|\varepsilon \right|}^{2}=0.99999$ (i.e., $\langle \hat{n}\rangle \approx 10^{5}$), we have $t_{n}=n{\left|\varepsilon \right|}^{2n}<0.001$ if only $n>2.2\times 10^{6}$. However, an approximate asymptotical behavior of functions (55) and (56) can be easily found if one takes into account that a consequence of Equations (23)–(24) and (55)–(56) is the formula

(59) $$D\left(|\varepsilon |\right)=\sigma_{x}^{min}\left(|\varepsilon |\right)\sigma_{x}^{max}\left(|\varepsilon |\right).$$

On the other hand, for r=±1, when $\langle \hat{x}\rangle =\langle \hat{p}\rangle =0$, we have the relations

(60) $$\sigma_{x}^{min}\left(|\varepsilon |\right)=\frac{1}{2}+\langle \hat{n}\rangle -\left|\langle {\hat{a}}^{2}\rangle \right|,\;\sigma_{x}^{max}\left(|\varepsilon |\right)=\frac{1}{2}+\langle \hat{n}\rangle +\left|\langle {\hat{a}}^{2}\rangle \right|.$$

Since $\sigma_{x}^{min}\left(|\varepsilon |\right)\to 0$ when |ε|→1, in this limit, we can write

$$\frac{1}{2}+\langle \hat{n}\rangle \approx \left|\langle {\hat{a}}^{2}\rangle \right|,\;\sigma_{x}^{max}\left(|\varepsilon |\right)\approx 1+2\langle \hat{n}\rangle .$$

Taking into account Equation (48), we arrive at the following asymptotic expression:

(61) $$D_{r}\left(|\varepsilon |\right)\approx \frac{1}{4}\ln\left(1+\langle \hat{n}\rangle \right)-2rS,\;r=\pm 1,\;\ln\left(1+\langle \hat{n}\rangle \right)\gg 1.$$

The difference $\Delta D=D_{od}-D_{ev}$ remains finite when $\langle \hat{n}\rangle \to \infty$ (contrary to the case of usual coherent states): ΔD≈4rS≈1.6.

## 5. The Mandel Factor

The Mandel factor [36]

(62) $$Q=\frac{\sigma_{n}-\langle \hat{n}\rangle }{\langle \hat{n}\rangle }=\frac{\langle {\hat{a}}^{†2}{\hat{a}}^{2}\rangle -{\langle {\hat{a}}^{†}\hat{a}\rangle }^{2}}{\langle {\hat{a}}^{†}\hat{a}\rangle }$$

can be easily calculated analytically, both for superpositions (6) and superpositions (8).

### 5.1. Usual Coherent States

**Figure 4:**
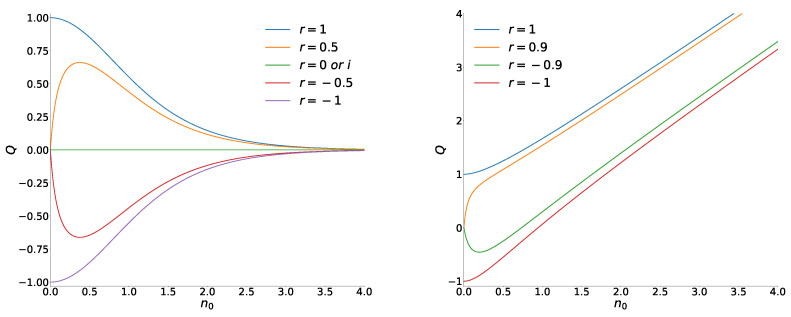
The Mandel factors of superpositions of coherent states (**left**) and coherent phase states (**right**) as functions of the mean number of quanta $n_{0}={\left|\alpha \right|}^{2}$ in the original coherent state (1) and $n_{0}={\left|\varepsilon \right|}^{2}/\left(1-{\left|\varepsilon \right|}^{2}\right)$ in the original coherent phase state (3) for different values of parameter *r*.

For superpositions (6), we obtain

(63) $$Q_{r\alpha }=\frac{4R\chi_{\alpha }{\left|\alpha \right|}^{2}}{1-R^{2}\chi_{\alpha }^{2}},\;R=\frac{2Re(r)}{1+{\left|r\right|}^{2}},$$

where $\chi_{\alpha }$ is defined in Equation (7). Note the (anti)symmetry property $Q_{r\alpha }=-Q_{-r\alpha }$. The Mandel factor equals zero not only for the coherent states but for their Yurke–Stoler superpositions as well. Asymptotically, $Q_{r\alpha }\approx 4R{\left|\alpha \right|}^{2}\exp\left(-{2|\alpha |}^{2}\right)$ for |α|≫1. If |α|≪1, then $Q_{r\alpha }\approx 4R{\left|\alpha \right|}^{2}/\left(1-R^{2}\right)$, provided $R^{2}\neq 1$. For the exceptional even and odd superpositions, we have another behavior: $Q_{\pm 1\alpha }\approx \pm \left(1-\frac{2}{3}{\left|\alpha \right|}^{4}\right)$ at |α|≪1. All these features are clearly seen on the left-hand side of Figure 4.

### 5.2. Coherent Phase States

The general formula for superpositions (8) has the form

(64) $$Q_{r\varepsilon }=\frac{n_{0}\left[1+R^{2}\chi_{\varepsilon }^{4}+2R\chi_{\varepsilon }\left(1+\chi_{\varepsilon }+\chi_{\varepsilon }^{2}\right)\right]}{\left(1-R\chi_{\varepsilon }^{2}\right)\left(1+R\chi_{\varepsilon }\right)}.$$

Here, $\chi_{\varepsilon }$ was defined in Equation (9). It can also be written in terms of the mean number of quanta in the coherent phase state

(65) $$\chi_{\varepsilon }=\frac{1}{1+2n_{0}},\;n_{0}=\frac{{\left|\varepsilon \right|}^{2}}{1-{\left|\varepsilon \right|}^{2}}.$$

We see that $Q_{r\varepsilon }=n_{0}$ (as for the thermal states), if R=0 (in particular, for the single coherent phase states and the Yurke–Stoler superpositions). The asymptotical behavior for $n_{0}\gg 1$ is given by the formula $Q\approx n_{0}+R/2$. For $n_{0}\ll 1$, we obtain $Q_{ev}\approx 1+2n_{0}^{2}$ if R=1, while $Q_{od}\approx -1+4n_{0}^{2}$ if R=−1. If |R|≠1, the Mandel factor goes to zero when $n_{0}\to 0$. However, its behavior is different for positive and negative values of parameter *R*. If Im(r)=0 and |r|≠1, then

(66) $$Q_{r\varepsilon }\approx n_{0}\left[2{\left(\frac{1+r}{1-r}\right)}^{2}-{\left(\frac{1-r}{1+r}\right)}^{2}\right],\;n_{0}\ll 1.$$

Illustrations of the function $Q_{r\varepsilon }\left(n_{0}\right)$ are given on the right-hand side of Figure 4.

## 6. Conclusions

We have compared the most popular measures of “non-classicality” – the degree of squeezing and the Mandel factor – in two families of quantum superposition states. The strongest squeezing can be observed in the even superpositions. In the case of coherent phase states, the minimal quadrature variance goes to zero in all three basic kinds of superpositions: even, odd, and Yurke–Stoler ones, when the mean number of quanta goes to infinity. For small mean numbers of quanta, the squeezing effect is stronger for the Yurke–Stoler superpositions, both for the usual and coherent phase states.

Significant differences are observed also in the behavior of the Mandel factor. In the case of usual coherent states, the type of statistics (sub- or super-Poissonian) does not depend on the mean photon number in the initial coherent state $n_{0}$, and the *Q*-factor tends to zero with an exponential accuracy when $n_{0}\to \infty$, for all superpositions. On the other hand, the sub-Poissonian statistics of superpositions of the coherent phase states is observed for odd superpositions with small mean photon numbers. For high mean photon numbers, the statistics is super-Poissonian for all kinds of superpositions. It is interesting that the difference $Q_{ev}-Q_{od}$ tends to the nonzero (unit) value if $n_{0}\to \infty$. In this limit, $Q∼n_{0}$, almost as in the thermal quantum states. Another interesting feature of the Q-factor is that this factor tends to zero when α→0 or ε→0 for all superpositions, except for two distinguished special cases: Q(0)=±1 for r=±1. If the coefficient *r* is close to ±1, the functions $Q_{r}\left(\alpha \right)$ or $Q_{r}\left(\varepsilon \right)$ rapidly become very close to the corresponding exceptional functions $Q_{\pm 1}\left(\alpha \right)$ or $Q_{\pm 1}\left(\varepsilon \right)$. If *r* is a pure imaginary number, the photon statistics in superpositions coincides with that of the initial coherent states (or coherent phase states).

In the case of superpositions of usual coherent states, each component is described by the Gaussian wave function. It is known that the Robertson–Schrödinger uncertainty product does not depend on the argument α for all Gaussian states. However, this product increases approximately as $\langle \hat{n}\rangle ={\left|\alpha \right|}^{2}$ in the case of superpositions with ${\left|\alpha \right|}^{2}\gg 1$. On the other hand, the RS uncertainty product in superpositions of coherent phase states (where each component is non-Gaussian) grows much more slowly (approximately logarithmically) as the function of the mean number of quanta $\langle \hat{n}\rangle ∼{\left(1-{\left|\varepsilon \right|}^{2}\right)}^{-1}$.

## Data Availability

Data are contained within the article.
